# Comparison of Spatial Modelling Approaches on PM_10_ and NO_2_ Concentration Variations: A Case Study in Surabaya City, Indonesia

**DOI:** 10.3390/ijerph17238883

**Published:** 2020-11-29

**Authors:** Liadira Kusuma Widya, Chin-Yu Hsu, Hsiao-Yun Lee, Lalu Muhamad Jaelani, Shih-Chun Candice Lung, Huey-Jen Su, Chih-Da Wu

**Affiliations:** 1 Department of Geomatics, National Cheng Kung University, Tainan City 70101, Taiwan; liadira13@mhs.geodesy.its.ac.id; 2 Department of Geomatics Engineering, Institut Teknologi Sepuluh Nopember, Surabaya City 60111, Indonesia; lmjaelani@geodesy.its.ac.id; 3 Department of Safety, Health, and Environmental Engineering, Ming Chih University of Technology, New Taipei City 24301, Taiwan; gracecyhsu@mail.mcut.edu.tw; 4 Department of Leisure Industry and Health Promotion, National Taipei University of Nursing and Health Sciences, Taipei City 112303, Taiwan; hsiaoyun07@ntunhs.edu.tw; 5 Research Center for Environmental Changes, Academia Sinica, Taipei City 11529, Taiwan; sclung@rcec.sinica.edu.tw; 6 Department of Atmospheric Sciences, National Taiwan University, Taipei City 10617, Taiwan; 7 Institute of Environmental Health, National Taiwan University, Taipei City 100025, Taiwan; 8 Department of Environmental and Occupational Health, National Cheng Kung University, Tainan City 70101, Taiwan; hjsu@mail.ncku.edu.tw; 9 National Institute of Environmental Health Sciences, National Health Research Institutes, Miaoli County 35053, Taiwan

**Keywords:** geographic and temporal weighted regression (GTWR), geographically weighted regression (GWR), land-use regression (LUR), nitrogen dioxide (NO_2_), particulate matter (PM_10_)

## Abstract

Because of fast-paced industrialization, urbanization, and population growth in Indonesia, there are serious health issues in the country resulting from air pollution. This study uses geospatial modelling technologies, namely land-use regression (LUR), geographically weighted regression (GWR), and geographic and temporal weighted regression (GTWR) models, to assess variations in particulate matter (PM_10_) and nitrogen dioxide (NO_2_) concentrations in Surabaya City, Indonesia. This is the first study to implement spatiotemporal variability of air pollution concentrations in Surabaya City, Indonesia. To develop the prediction models, air pollution data collected from seven monitoring stations from 2010 to 2018 were used as dependent variables, while land-use/land cover allocations within a 250 m to 5000 m circular buffer range surrounding the monitoring stations were collected as independent variables. A supervised stepwise variable selection procedure was applied to identify the important predictor variables for developing the LUR, GWR, and GTWR models. The developed models of LUR, GWR, and GTWR accounted for 49%, 50%, and 51% of PM_10_ variations and 46%, 47%, and 48% of NO_2_ variations, respectively. The GTWR model performed better (R^2^ = 0.51 for PM_10_ and 0.48 for NO_2_) than the other two models (R^2^ = 0.49–0.50 for PM_10_ and 0.46–0.47 for NO_2_), LUR and GWR. In the PM_10_ model four predictor variables, public facility, industry and warehousing, paddy field, and normalized difference vegetation index (NDVI), were selected during the variable selection procedure. Meanwhile, paddy field, residential area, rainfall, and temperature played important roles in explaining NO_2_ variations. Because of biomass burning issues in South Asia, the paddy field, which has a positive correlation with PM_10_ and NO_2_, was selected as a predictor. By using long-term monitoring data to establish prediction models, this model may better depict PM_10_ and NO_2_ concentration variations within areas across Asia.

## 1. Introduction

It is well documented that exposure to ambient air pollution can lead to increased mortality and morbidity and a shortened life expectancy [1,2]. Among a variety of particles in the atmosphere, pollutants such as PM_10_ (coarse particulate matter with an aerodynamic diameter smaller than 10 μm) and nitrogen dioxide (NO_2_) are commonly used as indicators of ambient air pollution [3,4,5]. This is because these pollutants are believed to be associated with several acute and chronic health conditions [6]. Moreover, they have demonstrated associations with increased levels of mortality and morbidity in megacities [7,8,9,10]. As a developing country with several densely-populated industrial cities, Indonesia faces a significant challenge relating to worsening environmental quality resulting from increased air pollution [11]. A study conducted by Greenstone and Fan demonstrated that, in the last two decades, Indonesia has experienced dramatic changes in air quality [12]. The study confirmed that high levels of air pollution are now undermining Indonesians’ health and may continually reduce the average life expectancy of citizens. Furthermore, previous studies have confirmed that in Indonesia exposure to air pollution can worsen health conditions, including respiratory diseases and lung cancer, and even general health burdens. [13,14,15]. 

Exposure to air pollution is a major issue for residents of developing countries, and it arises due to rapid industrialization and urbanization [16,17]. Increased levels of air pollution can be attributed to population growth, which brings with it increased human activities and removal of green space [18,19]. According to the literature, fast-growing cities in Southeast Asia, the Middle East, and the Western Pacific Region are suffering from severe air pollution levels that are five to 10 times greater than the levels recommended by the World Health Organization (WHO), implying potential negative health impacts on local residents [2]. Take Indonesia as an example: Indonesia is undergoing a rapid transformation with a great percentage of the population moving from rural towns to urban cities in search of more lucrative jobs. Nonetheless, residents may lose several years of life if high levels of air pollution are present throughout their lifetime [20]. Public awareness regarding the negative impacts of air pollution in Indonesia remains low due to a lack of evidence by in-situ observation. Therefore, accurate methods to assess within-city variability of air pollution are necessary [21].

In connection with the air pollution estimation methods, several spatio-temporal modeling approaches have been developed in previous studies, including land-use regression (LUR), geographically weighted regression (GWR), and geographically and temporally weighted regression (GTWR) [22,23,24,25]. In LUR, a multiple linear regression model is developed, which then links the air pollution concentrations observed in the network to the most predictive environmental characteristics (e.g., traffic, industry) [24]. Previous studies have applied this method to estimate the spatial variations in exposure to pollution [26,27]. In recent years, researchers have developed various statistical approaches in order to deal with spatial issues in modeling air pollution. GWR was introduced as an extended traditional model fitted by least squares regression and can effectively deal with spatial heterogeneity and autocorrelation problems [28,29]. This method refers to local modeling techniques and fits a regression model at each geographic area based on neighbors within a specific bandwidth [30]. Furthermore, researchers have extended GWR to a temporal dimension for spatio-temporal modeling, which is named GTWR. GTWR can address spatial and temporal non-stationarities simultaneously by developing a weight matrix based on spatiotemporal distance [30]. This method expands the boundary of local modeling techniques and has been applied in various disciplines [31,32].

Given the paucity of air pollution information and the low levels of awareness in local residents in Indonesia, this study has two objectives: (1) to estimate spatio-temporal concentration of PM_10_ and NO_2_ by in situ observation and application of advanced methods, (2) to explore the determinants influencing the concentrations of air pollution. It is noted this study further applied GWR and GTWR to examine and verify the estimation in order to replicate accurate results in addition to LUR. To our knowledge, this is the first study estimating air pollution concentration in Indonesia by comparing these three advanced methods. The study results can be used as references for establishing policies or regulations focusing on air quality control.

## 2. Materials and Methods 

### 2.1. Study Area

**Figure 1:**
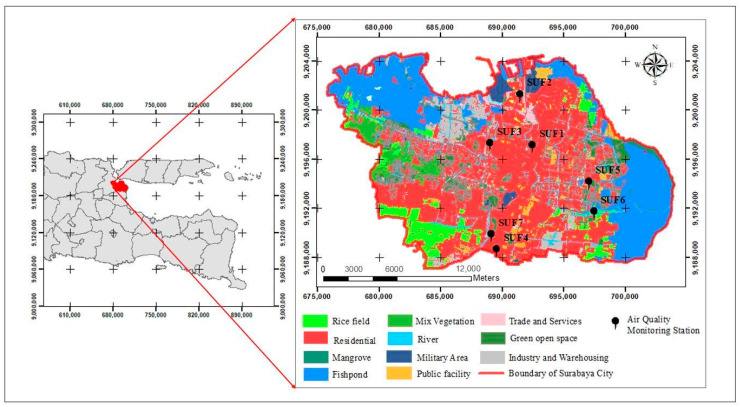
Location of Surabaya City and its land-use allocations.

Surabaya City (7°21′ S, 112°54′ E), the second largest city in Indonesia, was chosen as the study area. The size of Surabaya City is about 326.36 km^2^, which is divided into 31 districts and 154 villages. The northern and eastern portions of the city are surrounded by Madura Bay, while neighboring Sidoarjo County is in the southern portion and Gresik County is in the western portion. The population density is 8463 people/km^2^ [33]. Figure 1 illustrates most of Surabaya City was residential areas in 2014 [34]. Surabaya fixed station (SUF) 1, 4, and 7 are in rural areas, SUF 2 and 3 are in proximity to industrial areas, and SUF 5 and 6 are in urban areas. The development and establishment of road networks has helped residents in the region to more easily travel from one portion to another [35]. The population in Surabaya has increased each year, and air pollution due to transportation and industrial activity, too, have increased. The transportation sector contributes nearly 60% of pollutants, and, specifically, are responsible for 25% of the carbon dioxide (CO_2_), 90% of the carbon monoxide (CO), and 50% of the nitrogen oxide (NOx) present. In 2016 there were 2,244,317 units of gasoline-fueled vehicles and 150,413 units of diesel-fueled vehicles [36]. This number of vehicles will act to increase NO_2_ and PM_10_ levels due to motor vehicle fumes and dust agitated from the road [37].

### 2.2. Air-Pollution Monitoring Database

The Environmental Bureau of Surabaya City monitors PM_10_ and NO_2_ concentrations in Surabaya City via seven automatic monitoring stations distributed within the study area, as shown in Figure 1. For the model analysis, we aggregated hourly concentration observations from 2010 to 2018 into annual averages for model analysis.

### 2.3. Spatial Databases

Daily climate data were collected by Meteorological, Climatological, and Geophysical Bureau (BMKG) in the East Java Province (http://dataonline.bmkg.go.id/home). The monitoring stations collect information, including temperature, wind direction, wind speed, relative humidity, solar radiation, and rainfall. It is noted that the meteorological monitoring stations and air quality monitoring stations are in different locations. Therefore, the inverse distance weighting (IDW) interpolation method was employed to estimate meteorological conditions at air quality monitoring stations.

Land-use data for Surabaya City were provided by City Development Planning Bureau of Surabaya City (BAPPEKO) with its most recent update in 2014. As shown in Figure 1, the land use in Surabaya City was dominated by 47.85% residential area (red color), followed by 20.4% fishpond, 7.95% green open space, 6.04% paddy field, 5.35% industry and warehousing, and 2.79% public facility [34]. The land-use map used WGS 84 UTM Zone 49S as its coordinate reference system.

A greenness inventory can be obtained by satellite imagery, allowing easy access to multi-temporal greenness [21]. The normalized difference vegetation index (NDVI) was incorporated to represent the surrounding greenness during the study period. The Terra Moderate Resolution Imaging Spectroradiometer (MODIS) (MOD13Q1) version 6 was applied to adopt NDVI for a spatial resolution of 250 m × 250 m (https://ladsweb.modaps.eosdis.nasa.gov/search/order/1). Moreover, the best pixel value from all satellite images was chosen in the 16-day period based on the criteria of low clouds, low view angle, and the highest NDVI/enhanced vegetation index (EVI) value, as set by Didan [38]. There were two greenness values of NDVI for each month. We picked the one which was closest to the middle of the month (date, 15th) and then aggregated those for the annual average of each year. The greenness and all land use variables were abstracted from 250 m to 5000 m, with 250 m interval circular buffer ranges surrounding each PM_10_ and NO_2_ measurement site, representing the land-use/land cover allocations in the neighborhoods. Table S1 lists the potential predictor variables used in this study.

### 2.4. Model Developments Using Three Approaches and Validation

Numerous studies have presented LUR, GWR, and GTWR as methods suitable for investigating air pollution exposure, comparing the performance of the three methods in their analyses. For example, by using data from 56 monitoring stations, a study in Heilongjiang, China, applied several global and geographically-temporally weighted regression models to investigate PM_2.5_ related to O_2_, NO_2_, PM_10_, CO, and O_3_ [30]_._ The results demonstrated that temporally weighted regression (TWR) and GTWR yielded slightly better model performance than least square regression and GWR, indicating there are impacts on spatio-temporal variation in air quality. A study conducted in Eastern China also confirmed GTWR obtained the highest model performance in estimating ground-level PM_2.5_ concentrations compared to least square regression and GWR [23]. Furthermore, a recent study by Zeng and colleagues performed GTWR coupled with kriging-based hybrid models by applying three geospatio-temporal modeling approaches to analyze air quality data from specific industrial monitoring stations in Taiwan. It concluded GWTR had the best performance for predicting PM_10_ and O_3_ concentrations compared to LUR and GWR methods [39].

In this study, the land-use regression (LUR) models were built based on methodologies developed and verified in previous studies [16,25,26]. The first steps to develop the LUR model determined all parameters with the calculated pollutant as a dependent variable according to the strength of each association. Then, statistical analyses using Spearman correlation were used to look at the bivariate correlation between ambient air pollution and the three types of variable, such as land use, meteorological, and greenness variables. After that, variables that have absolute correlation to a high factor are kept in each sub-category (Spearman’s r ≥ 0.4). Then, all selected variables are entered into a stepwise linear regression. A supervised stepwise procedure was applied to increase the percentage of justified variability. For all potential predictor variables, we chose an a priori direction of effect to PM_10_ and NO_2_ concentrations (e.g., positive for road length and residential area, negative for NDVI and green spaces) [21,22,25]. The initial model included variables with the highest explained variance in a univariate analysis and the regression slope with the expected direction. After that, all other variables were added to the model separately, so as to assess whether or not the p-value was less than 0.1 and whether or not the variance inflation factor (VIF) was less than three. This procedure was repeated until none of variables met the criteria listed. Finally, R^2^, adjusted R^2^, and root mean square error (RMSE) were applied to evaluate the model performance.

The equation of the developed LUR model is defined as follows (1):

(1) $$Y=\beta_{0}+\beta_{1}X_{1}+\beta_{2}X_{2}+\ldots +\beta_{n}X_{n}\;\ldots \;$$

where *Y* is concentration of PM_10_ or NO_2_; *β*_0_ is the constant intercept; *β*_1_ to *β_n_* are regression coefficients; and *X*_1_ … *X_n_* are potential predictors.

In the second step, each of the selected variables was further examined by GWR and GTWR for developing the prediction models. GWR is an approach applicable for solving a model based on the spatial variation of parameters by region [40]. The GWR was created to extend the traditional global model fitted by ordinary least squares and can efficiently address spatial heterogeneity and autocorrelation issues [28,41]. The equation of the GWR model is defined as follows (2): an extension of the linear regression model, which allows researchers to bring data from surrounding samples to each region and establish individual regression models for which parameters vary by region,

(2) $$Y_{i}=\beta_{\left(U_{i},v_{i}\right)}+\sum_{k}^{p}\beta_{k\left(U_{i},\;v_{i}\right)}X_{ik}\ldots$$

where ($U_{i}$, $v_{i}$) denotes the coordinates of the point in location; $Y_{i}$ is concentration of PM_10_ or NO_2_; $\beta_{\left(U_{i},v_{i}\right)}$ represents the intercept; $\beta_{k\left(U_{i},v_{i}\right)}\;$ is a set of values of parameters at point  i; and $X_{ik}$ are potential predictors.

The GTWR model is an approach suitable for simultaneously solving non-stationarity spatial and temporal data. The equation of the GTWR model is defined as follows [42] (3):

(3) $$Y_{i}=\beta_{\left(U_{i},v_{i},t_{i}\right)}+\sum_{k}^{p}\beta_{k\left(U_{i},v_{i},t_{i}\right)}X_{ik}\;\ldots$$

where ($U_{i},\;v_{i},\;t_{i}$) denotes the coordinates of the points in space-time; $Y_{i}$ is concentration of PM_10_ or NO_2_; $\beta_{\left(U_{i},\;v_{i},\;t_{i}\right)}$ represents the intercept; $\beta_{k\left(u_{i},\;v_{i},\;t_{i}\right)}$ is a set of values of parameters at point  i; and $X_{ik}$ are potential predictors. The bandwidth value was selected by utilizing a corrected Akaike information criterion (AIC) which used to analyze the model performance and accuracy [43]. The corrected AIC was used since this information criterion method is one of the most commonly applied goodness-of-fit criteria for model comparisons [30,43,44]. Moreover, as noted in Fotheringham’s study, AIC offers the advantage of being more general in application and, thus, can be used to assess, by considering the degree of freedom, whether a regression model can provide a better fit than a global model [28].

This study used LUR to identify important prediction variables. The variables selected by LUR were then used for the GWR and GTWR. The developed models from the three approaches were validated for verification accuracy. For purposes of cross-validation, 90% of the air pollution measurements were randomly selected for training the model and the other 10% of data were used to validate the model predictions. This procedure was repeated 10 times to ensure each measurement had been used as out-of-sample data for the model evaluation. All of the statistical analyses were conducted using SPSS version 20 packages and R statistical packages x64 3.5.2 software. The spatial analyses were performed using ArcGIS 10.5.

## 3. Results

### 3.1. Particulate Matter (PM_10_) and Nitrogen Dioxide (NO_2_) Concentrations of Surabaya

**Figure 2:**
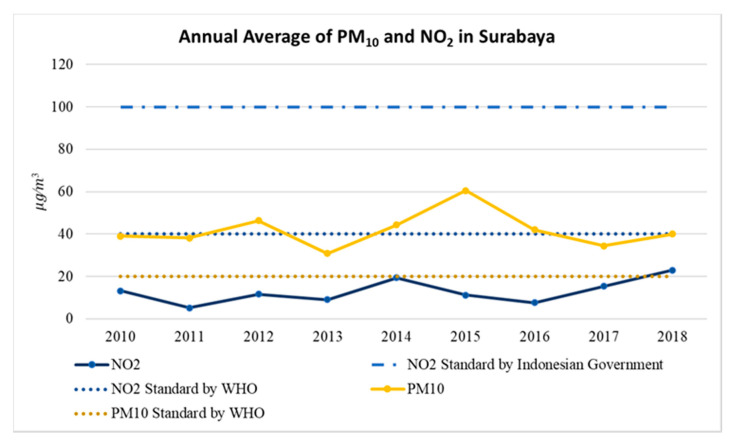
Time-series trend of air pollutants (particulate matter (PM_10_) and nitrogen dioxide (NO_2_) concentrations) in Surabaya from 2010 to 2018.

**Figure 3:**
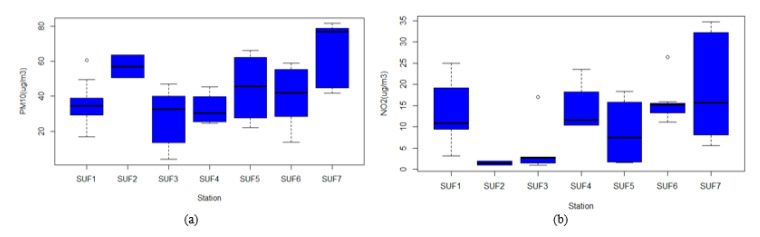
Box plots of (**a**) PM_10_ concentrations (**b**) NO_2_ concentrations of the 7 monitoring stations.

Figure 2 shows the annual trend of PM_10_ and NO_2_ concentrations calculated from information collected in all stations from 2010 to 2018 in Surabaya. Regarding PM_10_, the annual mean concentration of PM_10_ in Surabaya was 41.31 μg/m^3^, well beyond the 20 μg/m^3^ limit recommended by WHO [29]. The highest level was observed in 2015 (60.46 μg/m^3^) and the lowest level was recorded in 2013 (30.85 μg/m^3^). Meanwhile, the annual mean concentration of NO_2_ during the study period was 12.86 μg/m^3^. The highest concentration of NO_2_ was observed in 2018 (22.91 μg/m^3^) and the lowest concentration was detected in 2011 (5.2 μg/m^3^) (Figure 2). Overall, the annual level of NO_2_ did not change significantly during the 9-year period and did not exceed the 40 μg/m^3^ limit recommended by WHO or the 100 μg/m^3^ limit recommended by the Indonesian government. Figure 3 illustrates the boxplots of PM_10_ and NO_2_ concentrations at the seven monitoring stations. The highest concentrations of PM_10_ and NO_2_ were both observed in SUF7 (64.77 ± 19.72 μg/m^3^ for PM_10_ and 19.24 ± 13.49 μg/m^3^ for NO_2_) and the lowest concentration was recorded in SUF3 (27.502 ± 18.095 μg/m^3^ for PM_10_ and 5.021 ± 6.782 μg/m^3^ for NO_2_).

### 3.2. Model Developments and Validation

Table 1 lists the coefficients of selected variables with their respective p values, variance inflation factor (VIF), and partial R^2^ of LUR models developed in this study. For PM_10_ the final LUR model includes predictors such as public facility with a 5000 m radius, industry and warehousing within a 500 m radius, paddy field within a 2500 m radius, and NDVI within a 250 m radius, and their values, which were 0.10, 0.11, 0.12, and 0.17 partial R^2^, respectively. The results demonstrate PM_10_ to be positively related to proximity to a public facility, industrial and warehousing area, and paddy field. In contrast, PM_10_ had a negative relationship with NDVI. Moreover, NDVI accounted for 17% of PM_10_ variation and, thus, is the dominant variable for the developed model.

Regarding NO_2_, the predictors of paddy field within a 4250 m radius, residential area within a 4000 m radius, rainfall, and temperature were selected in the LUR model, yielding values of 0.16, 0.15, 0.08, and 0.06 partial R^2^, respectively. The concentration of NO_2_ was positively correlated with proximity to paddy field, proximity to residential area, and temperature, but it was negatively correlated with rainfall. The paddy field variable accounted for 16.4% of NO_2_ variation and, thus, is the dominant variable in the model.

Table 2 and Table 3 both display comparison results of the three different methods for PM_10_ and NO_2_, respectively, based on R^2^, adjusted R^2^, and Akaike information criterion (AIC). It is noted every selected variable was correlated with PM_10_ and NO_2_ in the same direction in the GWR and GTWR models as those in LUR. In Table 2, GWR model and GTWR model have higher values of R^2^ and lower values of AIC, indicating their model performances were better than the LUR model. Similarly, GWR model and GTWR model also show higher values of R^2^ and lower values of AIC than the LUR model when comparing models for NO_2_ (Table 3), indicating better performances for GWR and GTWR. Among the three approaches, GTWR-based models had the best prediction performance and a moderate explanatory power. As for model validation, the 10-fold cross validation R^2^ were 0.52 and 0.53 for the GTWR-based models for PM_10_ and NO_2_, respectively. The 10-cross validation confirms the robustness of the developed prediction models.

### 3.3. PM_10_ and NO_2_ Concentrations Variations

**Figure 4:**
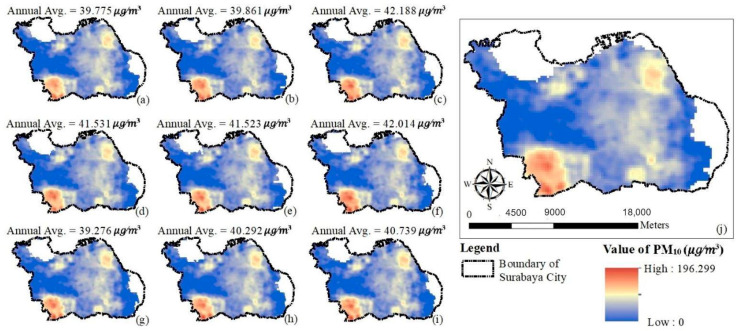
Prediction maps of PM_10_ concentration variations using GWR model: (**a**) 2010 (**b**) 2011 (**c**) 2012 (**d**) 2013 (**e**) 2014 (**f**) 2015 (**g**) 2016 (**h**) 2017 (**i**) 2018 (**j**) average from 2010 to 2018.

**Figure 5:**
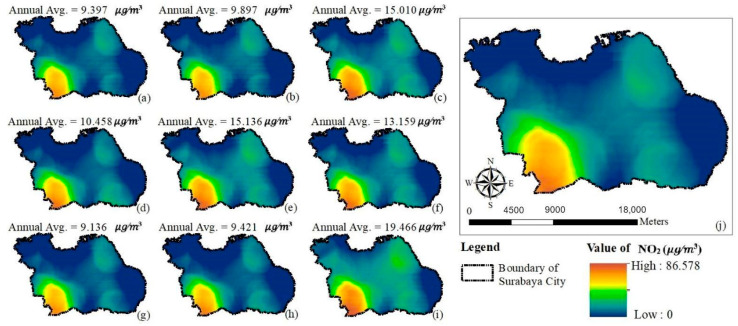
Prediction maps of NO_2_ concentration variations using GWR model: (**a**) 2010 (**b**) 2011 (**c**) 2012 (**d**) 2013 (**e**) 2014 (**f**) 2015 (**g**) 2016 (**h**) 2017 (**i**) 2018 (**j**) average from 2010 to 2018.

Figure 4 and Figure 5 represent the annual average concentration of PM_10_ and NO_2_, respectively, for the entire study period when applying the GWR model. Blue to red and green to red on the maps represent the respective levels from low to high of PM_10_ and NO_2_ concentrations. Both figures illustrate that the southwestern portion of Surabaya City had the highest PM_10_ and NO_2_ concentrations throughout the predicted period.

**Figure 6:**
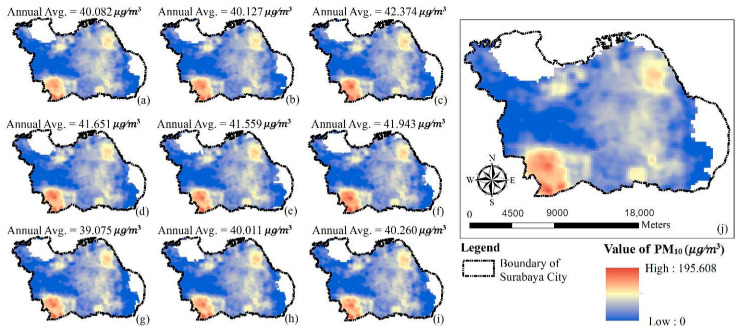
Prediction maps of PM_10_ concentration variations using GTWR model: (**a**) 2010 (**b**) 2011 (**c**) 2012 (**d**) 2013 (**e**) 2014 (**f**) 2015 (**g**) 2016 (**h**) 2017 (**i**) 2018 (**j**) average from 2010 to 2018.

**Figure 7:**
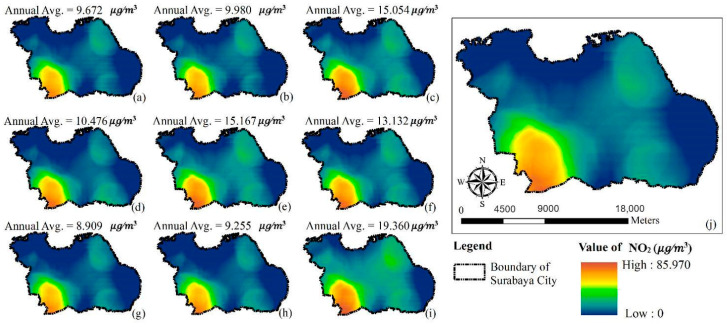
Prediction maps of NO_2_ concentration variations using GTWR model: (**a**) 2010 (**b**) 2011 (**c**) 2012 (**d**) 2013 (**e**) 2014 (**f**) 2015 (**g**) 2016 (**h**) 2017 (**i**) 2018 (**j**) average from 2010 to 2018.

Figure 6 and Figure 7 illustrate the annual average concentration of PM_10_ and NO_2_, respectively, for the entire study period, as simulated by the GTWR model. Blue to red and green to red on the maps represent the levels of respective PM_10_ and NO_2_ concentrations from low to high. Both figures illustrate the southwest portion of Surabaya City had the highest PM_10_ and NO_2_ concentrations throughout the predicted period.

**Figure 8:**
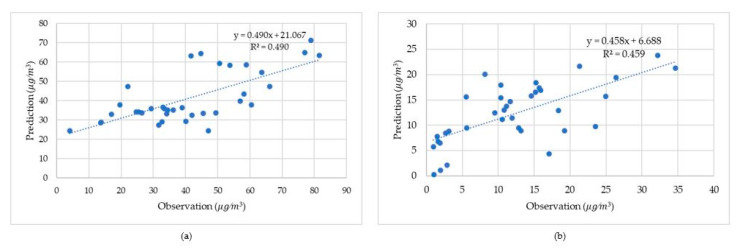
External verification using GWR based on observations regressed against predictions. (**a**) PM_10_ (**b**) NO_2._

Figure 8 displays the comparison using the GWR method between model predictions and on-site observations. The R^2^ was 0.49 for PM_10_ and 0.459 for NO_2_, with *p* values less than 0.05, confirming the robustness of established models.

**Figure 9:**
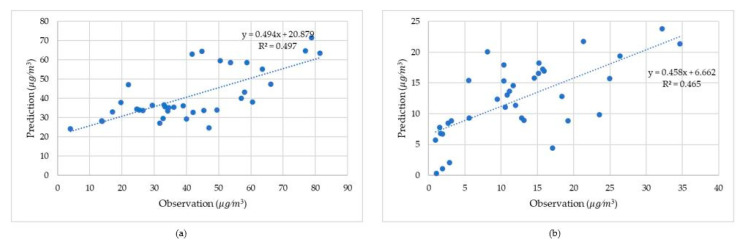
Verification using GTWR based on observations regressed against predictions. (**a**) PM_10_ (**b**) NO_2._

Figure 9 illustrates the comparison using the GTWR method between model predictions and on-site observations. The R^2^ was 0.497 for PM_10_ and 0.465 for NO_2_, with *p* values less than 0.05, confirming the robustness of established models.

## 4. Discussion

To the best of our knowledge, this is the first study to estimate PM_10_ and NO_2_ concentrations across Surabaya City, Indonesia, by employing three different methods (i.e., LUR, GWR and GTWR) with the use of land-use information and weather data in order to examine the determinants influencing air quality. According to the study results, land use type, (e.g., paddy fields, residential areas, industry and warehousing), climate conditions (e.g., rainfall and temperature), and greenness were determinants influencing air quality. The study results could be used for reference in developing countries similar to Indonesia for establishing policies or regulations focusing on air quality control. 

Several studies have demonstrated meteorological factors such as humidity, temperature, wind speed and wind direction are highly correlated with air pollution [45,46]. Consistent with these studies, our results reflected a positive relationship between temperature and NO_2_. In contrast, increased humidity and rainfall are protective factors against air pollution [47], which is consistent with the results of our study. In addition to meteorological factors that cannot be controlled, greenness plays a significant role in reduction of air pollutants [47,48]. Our study findings demonstrated greenness to have a protective effect against air pollutants. As such, it is suggested that local governments prioritize retaining a greater percentage of greenness when executing urban planning and design.

Consistent with previous studies [49,50,51,52], our results confirm that types of land use, such as public facility, industry and warehousing, residential area, and paddy field are positively associated with PM_10_ and NO_2_ levels. Prior studies have indicated that higher levels of NO_2_ may come from cooking fuels and biomass burning [49,50,53], helping to explain the relationship between air pollution and both residential areas and paddy fields. Therefore, it is suggested that every household should have an exhaust hood in order to cope with emissions of cooking oil fumes. Meanwhile, biomass burning should be banned in order to prevent producing an avoidable amount of harmful air pollutants. In addition, the insignificant impact from climate conditions (temperature and rainfall) on PM_10_ concentrations may be because other variables (e.g., paddy field and NDVI) influence the seasonal variation more than climate conditions.

According to the study results the southwest portion of Surabaya City has the highest PM_10_ and NO_2_ concentrations. This may be because the southwest portion of the country has the highest residential density and road traffic. According to the findings of a previous study focusing on Surabaya City, the air quality of the city is related to its traffic volume, seeing as air quality has continually worsened as the number of vehicles on the road has increased [46]. Hence, developing a public transportation system and encouraging the use of electrical vehicles may be ways to alleviate the traffic burden and to improve air quality.

This study made a comparison between three methods to examine the influence of determinants in PM_10_ and NO_2_ concentrations. Although solid analyses were employed, there are some limitations to this study. First, Surabaya only has seven monitoring stations and the monitoring stations are not evenly distributed throughout the city (e.g., there are no air pollution monitoring stations in the western portion of Surabaya City), reducing the generalizability of study results. However, the distribution of monitoring stations within a circular buffer covers a large percentage of the study site and was able to include major factors such as residential and industrial areas. Second, given that Surabaya City had more than two million vehicles in 2015 and the total number of vehicles steadily increased from 2011 to 2015 [54], traffic conditions should be included in the model. Third, using the IDW interpolation method to estimate meteorological conditions can result in specific errors. Nonetheless, there are no traffic intensity data available, limiting the degree of explanation of the final model. Lastly, the lack of MODIS aerosol optical depth is another limitation in this study since this was considered an important variable for estimating PM_10_ and NO_2_ [55]. While a moderate model predictive power was obtained from this study, we suggest future studies consider the aforementioned predictor variables in order to improve model prediction performance. 

## 5. Conclusions

This is the first study to implement spatio-temporal variations of pollution concentration in Surabaya City, Indonesia, by making a comparison between LUR, GWR, and GTWR models. The results show land-use type, climate conditions, and greenness were determinants influencing air quality. Moreover, by using data from a monitoring network which routinely monitors air quality with different characteristics in different areas, we confirmed that the models developed in this study can predict fine spatial variability in short-term and long-term outdoor PM_10_ and NO_2_ concentrations. This study helps us to better understand the air quality in Surabaya City and, in effect, helps to provide a direction for future epidemiological studies.

## Figures and Tables

**Table 1:** The developed land-use regression (LUR) model for PM_10_ and NO_2._

**PM_10_**				
**Variable**	**Coefficients**	p **-Value**	**VIF**	**Partial R^2^**
Intercept	36.28	<0.01	-	-
^a^ Public Facility_5000m_	0.562	<0.01	1.39	0.10
^b^ Industry and Warehousing_500m_	0.027	0.01	1.33	0.11
^c^ Paddy Field_2500m_	0.185	<0.01	2.22	0.12
^d^ NDVI_250m_	−191	<0.01	2.89	0.17
**NO_2_**				
**Variable**	**Coefficients**	p **-Value**	**VIF**	**Partial R^2^**
Intercept	−374.13	0.08	-	-
^e^ Paddy Field_4250m_	0.146	<0.01	2.70	0.16
^f^ Residential Area_4000m_	0.013	<0.01	1.90	0.15
Rainfall	−3.028	<0.01	1.71	0.08
Temperature	13.212	0.08	2.75	0.06

^a^ Public facility within a radius of 5000 m; ^b^ Industry and Warehousing within a radius of 500 m; ^c^ Paddy field within a radius of 2500 m; ^d^ Average normalized difference vegetation index (NDVI) within a radius of 250 m; ^e^ Paddy field within a radius of 4250 m; ^f^ Residential area within a radius of 4000 m.

**Table 2:** Comparison of Final Model for PM_10_ using LUR, geographically weighted regression (GWR) and geographic and temporal weighted regression (GTWR).

	LUR	GWR (Bandwidth: 1.989)	GTWR (Bandwidth: 1.414)
Intercept	36.28 ^a^	36.20–36.50 ^b^	35.90–37.50 ^b^
Public Facility_5000m_^c^	0.562	0.559–0.560	0.544–0.563
Industry and Warehousing_500m_^d^	0.027	0.0271–0.0272	0.025–0.029
Paddy Field_2500m_^e^	0.185	0.184–0.185	0.180–0.186
NDVI_250m_^f^	−191	−191.00~−190.00	−193~−186
R^2^	0.49	0.50	0.51
adjusted-R^2^	0.42	0.44	0.45
AIC	310.00	305.14	305.03

^a^ coefficient estimates; ^b^ minimum and maximum of the coefficient estimates; ^c^ Public facility within a radius of 5000 m; ^d^ Industry and Warehousing within a radius of 500 m; ^e^ Paddy field within a radius of 2500 m; ^f^ Average NDVI within a radius of 250 m.

**Table 3:** Comparison of final model for NO_2_ using LUR, GWR and GTWR.

	LUR	GWR (Bandwidth: 1.987)	GTWR (Bandwidth: 1.985)
Intercept	−374.13 ^a^	−377~−367 ^b^	−377~−366 ^b^
Paddy Field_4250m_^c^	0.146	0.145~0.146	0.144~0.146
Residential Area_4000m_^d^	0.013	0.0129~0.0131	0.0127~0.0131
Rainfall	−3.028	−3.07~−2.99	−3.06~−2.95
Temperature	13.212	12.9~13.3	12.90~13.30
R^2^	0.46	0.47	0.48
adj-R^2^	0.39	0.41	0.41
AIC	252.00	252.08	251.81

^a^ coefficient estimates; ^b^ minimum and maximum of the coefficient estimates; ^c^ Paddy field within a radius of 4250 m; ^d^ Residential area within a radius of 4000 m.

## Supplementary Materials

The following are available online at https://www.mdpi.com/1660-4601/17/23/8883/s1, Table S1: List of potential predictor variables collected from each database.
