# Supplementary material for: Comparison of Spatial Modelling Approaches on PM10 and NO2 Concentration Variations: A Case Study in Surabaya City, Indonesia
Source: Int J Environ Res Public Health. 2020 Nov 29;17(23):8883. doi: 10.3390/ijerph17238883 (PMC7730102; doi:10.3390/ijerph17238883)
Supplement: Supplementary file 1 [file ijerph-17-08883-s001.pdf]

Comparison of Spatial Modelling Approaches on PM_{10} and NO_2 Concentration Variations: A Case Study in Surabaya City, Indonesia

Liadira Kusuma Widya [1,2], Chin-Yu Hsu [3], Hsiao-Yun Lee [4], Lalu Muhamad Jaelani [2], Shih-Chun Candice Lung [5,6,7], Huey-Jen Su [8] and Chih-Da Wu [1,9,*].

[1] Department of Geomatics, National Cheng Kung University, 70101 Tainan City, Taiwan; liadira13@mhs.geodesy.its.ac.id (L.K.W.); chidawu@mail.ncku.edu.tw (C.D.W.)

[2] Department of Geomatics Engineering, Institut Teknologi Sepuluh Nopember, 60111 Surabaya City, Indonesia; liadira13@mhs.geodesy.its.ac.id (L.K.W.); lmjaelani@geodesy.its.ac.id (L.M.J.)

[3] Department of Safety, Health, and Environmental Engineering, Ming Chih University of Technology, 24301 New Taipei City, Taiwan; gracecyhsu@mail.mcut.edu.tw (C.Y.H.)

[4] Department of Leisure Industry and Health Promotion, National Taipei University of Nursing and Health Sciences, 112 Taipei City, Taiwan; hsiaoyun07@ntunhs.edu.tw (H.Y.L.)

[5] Research Center for Environmental Changes, Academia Sinica, 11529 Taipei City, Taiwan; sclung@rcec.sinica.edu.tw (S.C.C.L.)

[6] Department of Atmospheric Sciences, National Taiwan University, 10617 Taipei City, Taiwan; sclung@rcec.sinica.edu.tw (S.C.C.L.)

[7] Institute of Environmental Health, National Taiwan University, 100 Taipei City, Taiwan; sclung@rcec.sinica.edu.tw (S.C.C.L.)

[8] Department of Environmental and Occupational Health, National Cheng Kung University, 70101 Tainan City, Taiwan; hjsu@mail.ncku.edu.tw (H.J.S.)

[9] National Institute of Environmental Health Sciences, National Health Research Institutes, 35053 Miaoli County, Taiwan; chidawu@mail.ncku.edu.tw (C.D.W.)

* Correspondence: chidawu@mail.ncku.edu.tw

Table S1. List of potential predictor variables collected from each database.

Data Source	Potential variables	Geo-format	Measurement	Expected Association
**Air Pollutant Database**				
Environmental Bureau of Surabaya City	PM_{10}	point	On-site measurement (μg/m^3)	+
	NO_X	point	On-site measurement (ppm)	+
**Meteorological Database**				
Meteorological, Climatological, and Geophysical Bureau of Indonesia	Temperature	point	On-site measurement (°C)	-/+
	Relative humidity	point	On-site measurement (%)	-/+
	Wind speed	point	On-site measurement (km/h)	-/+
	Wind direction	point	On-site measurement	-/+
	Rainfall	point	On-site measurement (mm/d)	-/+
	Duration of solar radiation	point	On-site measurement (joule/cm^2)	-/+
**GIS – Land Use Database**				
City Development	Water reservoirs	point	Number within 250-5000m circular buffers (count)	-/+

Planning Bureau of Surabaya City (BAPPEKO)	Installation facility	point	Number within 250-5000m circular buffers (count)	-/+
	Sports facility	point	Number within 250-5000m circular buffers (count)	-/+
	Government facility	point	Number within 250-5000m circular buffers (count)	-/+
	Education facility	point	Number within 250-5000m circular buffers (count)	-/+
**Data Source**	**Potential variables**	**Geo-format**	**Measurement**	**Expected Association**
**GIS – Land Use Database**				
City Development Planning Bureau of Surabaya City (BAPPEKO)	Trading facility	point	Number within 250-5000m circular buffers (count)	-/+
	Worship facility	point	Number within 250-5000m circular buffers (count)	-/+
	Office facility	point	Number within 250-5000m circular buffers (count)	-/+
	Social facility	point	Number within 250-5000m circular buffers (count)	-+
	Terminal facility	point	Number within 250-5000m circular buffers (count)	-/+
	Public facility	point	Number within 250-5000m circular buffers (count)	-/+
	Tower	point	Number within 250-5000m circular buffers (count)	-/+

	PLN substation	point	Number within 250-5000m circular buffers (count)	-/+
	Cleanliness Facility	point	Number within 250-5000m circular buffers (count)	-/+
	Health facility	point	Number within 250-5000m circular buffers (count)	-/+
	Penitentiary facility	point	Number within 250-5000m circular buffers (count)	-/+
	Industry and Warehousing	polygon	Area within 250-5000m circular buffers (m^2)	+
	Military Area	polygon	Area within 250-5000m circular buffers (m^2)	-/+
	Mangrove	polygon	Area within 250-5000m circular buffers (m^2)	-
	Trade and Services	polygon	Area within 250-5000m circular buffers (m^2)	+

**Data Source**	**Potential variables**	**Geo-format**	**Measurement**	**Expected Association**
**GIS – Land Use Database**				
City Development Planning Bureau of Surabaya City (BAPPEKO)	Residential	polygon	Area within 250-5000m circular buffers (m^2)	+
	Major road	polygon	Area within 250-5000m circular buffers (m^2)	+
	Swamp	polygon	Area within 250-5000m circular buffers (m^2)	-

	Green open space	polygon	Area within 250-5000m circular buffers (m^2)	-
	Paddy field	polygon	Area within 250-5000m circular buffers (m^2)	-/+
	Shrubs	polygon	Area within 250-5000m circular buffers (m^2)	-/+
	River	polygon	Area within 250-5000m circular buffers (m^2)	-
	Fishpond	polygon	Area within 250-5000m circular buffers (m^2)	-
	Moor	polygon	Area within 250-5000m circular buffers (m^2)	-/+
**Satellite Database**				
MODIS Greenness Database	NDVI	grid	Area within 250-5000m circular buffers (dimensionless)	-
